# Autoimmune/inflammatory syndrome induced by adjuvants in a woman with Hashimoto thyroiditis and familial autoimmunity—a case report and literature review

**DOI:** 10.3389/fimmu.2023.1139603

**Published:** 2023-05-23

**Authors:** Aleksandra Plavsic, Snezana Arandjelovic, Milan Dimitrijevic, Natasa Kusic, Vesna Tomic Spiric, Bojana Popovic, Zikica Jovicic, Aleksandra Peric Popadic, Rada Miskovic

**Affiliations:** ^1^ Clinic for Allergy and Immunology, University Clinical Centre of Serbia, Belgrade, Serbia; ^2^ Faculty of Medicine, University Clinical Centre of Serbia, Belgrade, Serbia; ^3^ Clinic for Endocrinology, Diabetes, and Metabolic Diseases, University Clinical Centre of Serbia, Belgrade, Serbia

**Keywords:** ASIA, Hashimoto thyroiditis, familial autoimmunity, AID, SBI

## Abstract

**Introduction:**

Autoimmune/inflammatory syndrome induced by adjuvants (ASIA) consists of a wide spectrum of symptoms and immunological features that are believed to develop in predisposed individuals after exposure to an adjuvant, including a silicone breast implant (SBI). Different autoimmune diseases (AIDs) have been associated with ASIA, but ASIA development after SBI in women with Hashimoto thyroiditis (HT) and familial autoimmunity has rarely been described.

**Case report:**

A 37-year-old woman presented in 2019 with arthralgia, sicca symptoms, fatigue, + antinuclear antibody (ANA), + anti SSA, and + anticardiolipin Immunoglobulin G (IgG) antibodies. She was diagnosed with HT and vitamin D deficiency in 2012. The familial autoimmunity was present: the patient’s mother had been diagnosed with systemic lupus erythematosus and secondary Sjogren’s syndrome and her grandmother with cutaneous lupus and pernicious anemia. In 2017, the patient had a cosmetic SBI procedure that was complicated by repeated right breast capsulitis. After 2 years of irregular visits due to COVID-19, she presented with + ANA, + anticentromere antibodies both in sera and seroma, sicca syndrome, arthralgias, twinkling in extremities, abnormal capillaroscopic findings, and reduced diffusing capacity of the lungs for carbon monoxide. She was diagnosed with ASIA, and antimalarial and corticosteroid therapy were introduced.

**Conclusion:**

In patients with HT and familial autoimmunity, SBI should be carefully considered due to the possibility of ASIA development. Hashimoto thyroiditis, familial autoimmunity, and ASIA seem to be interconnected in the complex mosaic of autoimmunity in predisposed individuals.

## Introduction

Autoimmune/inflammatory syndrome induced by adjuvants (ASIA) was described by Schoenfeld and Agmon-Levin and consists of heterogeneous clinical features and immunological dysfunction triggered by exposure to an adjuvant in genetically predisposed individuals (1). Since its introduction back in 2011, a lot of data have been collected and new insights into this intriguing subject have emerged. In genetically predisposed women, SBI can stimulate the immune system causing different symptoms and ultimately autoimmune disease (AID) (2). Many reports found the association between AID and ASIA (3–5). Whereas few studies found no association with connective tissue disease (CTD) (6), many physicians are not convinced that the symptoms perceived by the patients are caused by their breast implants. These conflicting data have caused an ongoing debate about the safety of SBI considering that this is a very popular aesthetic procedure worldwide. The question of safety is particularly important in those women who are already diagnosed with AID, such as Hashimoto thyroiditis (HT).

HT is an organ-specific AID characterized by autoimmune-triggered inflammation, lymphocyte infiltration, progressive destruction of the thyroid gland, and the presence of antibodies specific to thyroid antigens. It is the most common AID, with a prevalence of 7.5% (7). Given its high prevalence, it is important to assess the risk factors that could lead to the development of additional AID and/or ASIA. HT has been described in association with several other AIDs such as diabetes mellitus type 1, celiac disease, multiple sclerosis, systemic lupus erythematosus (SLE), autoimmune hepatitis, sarcoidosis, chronic urticaria, rheumatoid arthritis, and possibly myasthenia gravis (8). In a large, prospective study of 3,069 consecutive patients with chronic autoimmune thyroiditis, a high prevalence of other AIDs was found, autoimmune gastritis being the most frequent one (9). The association of HT with autoimmune atrophic gastritis and celiac disease presenting an example of polyautoimmunity and pathophysiological mechanisms of this thyro-entero-gastric autoimmunity link include common embryological origin, genetic susceptibility, and environmental factors (10). The altered intestinal microbiota composition and Breg cells also play a role in the pathogenesis of HT and polyautoimmunity (11, 12). The explanations for relationship between thyroid autoimmunity and other AIDs were examined in an article by Szyper-Kravitz et al., and they proposed the following: antithyroid antibodies’ immunomodulatory effect, molecular mimicry between thyroid and disease-specific epitopes, and a genetic link between autoimmunity and AID susceptibility (8).

Autoimmune diseases, including organ-specific and systemic ones are characterized by complex interactions between multiple genetic, immunological, environmental, and hormonal factors leading to inflammation and production of autoantibodies in predisposed individuals (13, 14). There are a couple of terms that are used to describe the complexity and connectivity of different AIDs. Polyautoimmunity refers to the coexistence of two or more AIDs in a single patient (15). Familial autoimmunity is defined as the presence of diverse autoimmune diseases in multiple members of a nuclear family (15). Familial autoimmune disease refers to the presence of one specific AID in various members of a nuclear family (15). The mosaic of autoimmunity describes the diversity of autoimmune manifestations and multifactorial origins in susceptible individuals and implies different combinations of numerous factors involved in autoimmunity (8, 16, 17). All these terms are used to indicate that despite the heterogeneity and diversity in the pathological, epidemiological, and clinical presentation of different AIDs in one individual or in members of a nuclear family, there might be similarities in genetic and environmental characteristics shared by the affected individual/family members. This interconnection of AID is especially important in order to evaluate the factors that can be assessed, corrected, or avoided in order to prevent the possibility of the AID.

Here, we present a young woman with HT and a familial history of autoimmune diseases who developed ASIA after SBI.

## Case report

**Figure 1:**
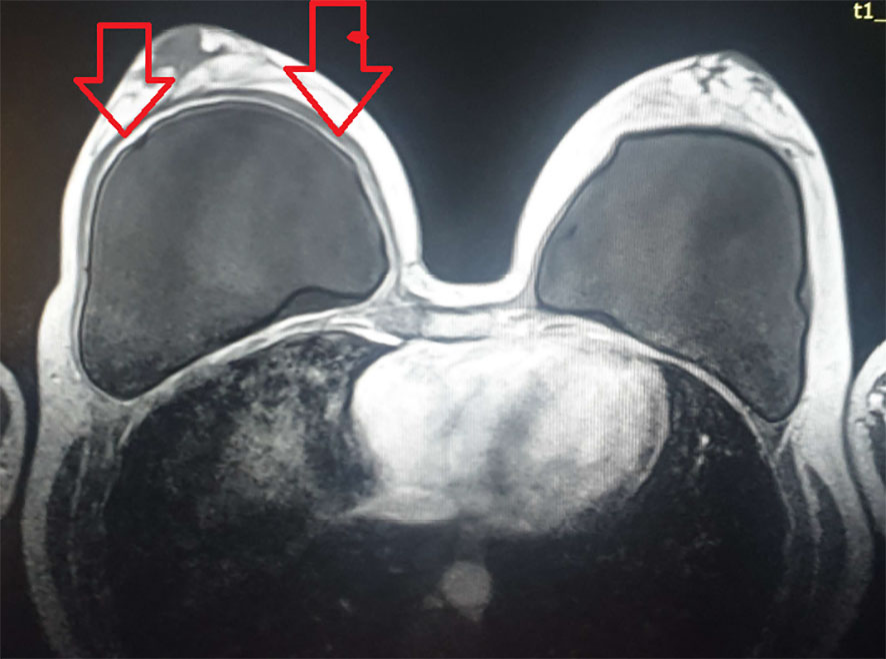
MRI after a silicone breast implant (SBI) showing the signs of right breast capsulitis. MRI, magnetic resonance imaging; SBI, silicone breast implant.

A 37-year-old woman first presented in the beginning of 2019 complaining of arthralgias, sicca symptoms, tiredness, and fatigue. Her personal history revealed that she was a non-smoker without prior allergies. She had been diagnosed with HT and vitamin D deficiency since 2012, but she was taking vitamin D irregularly. Family history suggested a strong autoimmune disease background: her mother had SLE and Sjogren’s syndrome, and her grandmother had cutaneous lupus and pernicious anemia. In 2017, the patient had a cosmetic SBI. However, a seroma was found repeatedly in the right breast that required drainage on three occasions. At that point, no seroma analysis was performed. She was treated by a plastic surgeon and periodically received short corticosteroid courses and antibiotics. Prednisone therapy led to some symptoms’ relief and seroma reduction, but the symptoms re-emerged after the corticosteroid therapy was discontinued. In 2018, magnetic resonance imaging (MRI) showed right breast capsulitis; a year later, breast ultrasound showed no signs of rupture and contractures (**Figure 1**).

At her first visit at our clinic in 2019, her erythrocyte sedimentation rate was 48 mm/h, complete blood count, CRP, D-dimer, and complete biochemistry (Blood urea nitrogen (BUN), creatinine, Na, K, Cl, total protein, albumin, bilirubin, aspartate transferase (AST), ALT, alkaline phosphatase (ALP), gamma-glutamyl transferase (GGT), creatine kinase (CK), and lactate dehydrogenase (LDH)) were normal, as well as urine analysis. The TSH and FT4 levels were in the reference range. The 25 (OH) vitamin D level was low—22 nmol/L indicating deficiency). The physical examination was unremarkable. Immunological analysis showed a positive ANA homogenous/nucleolar pattern on the Hep2 cells substrate in the titter 1:640, positive anticardiolipin (aCL) IgG, and anti-SSA antibodies. Ophthalmological testing was normal, as were nailfold capillaroscopy and abdominal ultrasound. The unstimulated salivary flow rate was 0.2 ml/min. The diagnosis of ASIA was made based on clinical presentation (arthralgias, sicca symptoms, tiredness, and fatigue), immunological tests (+ ANA, + anti SSA antibodies, and aCL IgG), the co-occurrence of AID (HT), a family history of AID, and time correlation between SBI and clinical presentation.

The removal of SBI was suggested, and vitamin D therapy was introduced. Also, salivary gland biopsy was indicated for suspected Sjogren’s syndrome and genetic testing as well. However, the tests were not completed due to the COVID-19 outbreak, and the patient missed her follow-up visits.

Almost 2 years since the first visit, she returned with similar symptoms, but some new ones have appeared in the meantime, such as skin and vaginal dryness and tingling in extremities. Also, hypothyroidism was diagnosed in 2020 and levothyroxine therapy was introduced. The seroma in the right breast was found once again. An ultrasound revealed right breast intracapsular rupture and contracture in 2021. The immunological analysis of seroma was positive for ANA (indirect immunofluorescence, IIF) of centromere pattern in the titer 1:640 and anticentromere (ELISA) antibodies. There were no malignant cells in seroma, and bacteriological and mycological findings were normal. Immunological testing in the serum showed + ANA on Hep-2 cell substrate (IIF) centromere pattern 1:640. Her laboratory work was normal, as well as her serum vitamin D level. In the following months, an extensive diagnostic workup was done. Keratoconjunctivitis, the unspecific nailfold capillaroscopic changes, and reduced diffusing capacity of the lungs for carbon monoxide (67%) were found. There were no abnormalities in her spirometry, chest CT, heart ultrasound, electroneuromyography, and color Doppler ultrasound of lower extremities.

**Figure 2:**
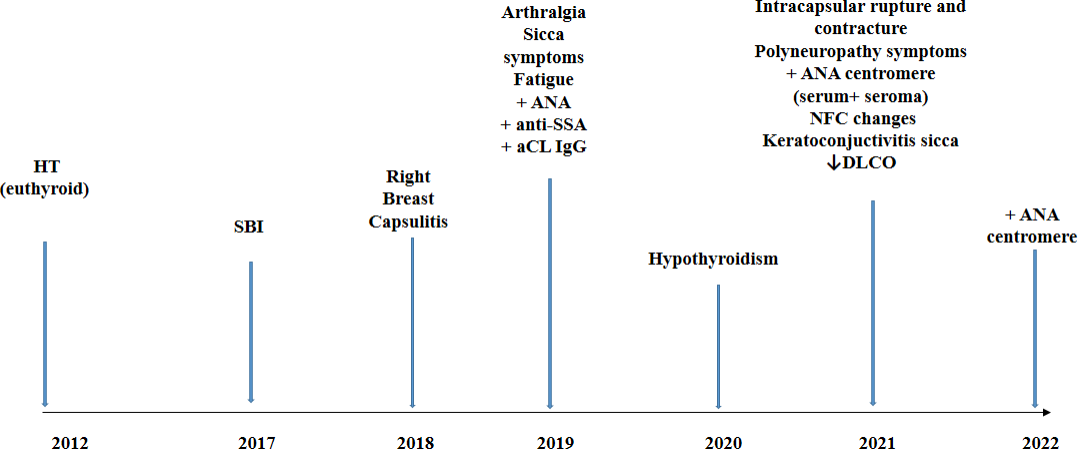
Timeline of the case report. HT, Hashimoto thyroiditis; SBI, silicone breast implant; aCL, anticardiolipin antibodies; NFC, nailfold capillaroscopy; DLCO, diffusing capacity of the lungs for carbon monoxide.

There was an evolution of the patient’s symptoms over the years, alongside with the change of autoantibodies profile from 2012 to 2021 as shown in **Figure 2**. We treated the patient with corticosteroid therapy (prednisone 20 mg daily initially with tapering) and antimalarial (hydroxychloroquine sulfate 2 × 200 mg), and vitamin D supplementation was continued. The therapy led to the reduction of most symptoms, except for the persistence of sicca symptoms. The explanation of SBI was indicated, but the patient was reluctant to accept it.

## Discussion

ASIA is characterized by heterogeneous clinical and immunological findings that are present in susceptible individuals after exposure to environmental immune stimulators (1). Many reports have found an association between AID and ASIA. In a study among 300 ASIA patients, 89% had clinically well-defined AID and 5.3% had a diagnosis of vasculitis (18). In a large cross-sectional study by Watad et al., among 24,651 women with SBI matched with 98,504 women without SBI, the strongest association was found between SBI and Sjogren’s syndrome, systemic sclerosis, and sarcoidosis (19). However, the literature has not so far addressed the possible connection between HT and ASIA extensively. In 32 consecutive patients with SBI and confirmed ASIA, 17 patients had systemic AID and 7 organ-specific AID, 3 of which had autoimmune thyroiditis (20). This is very interesting considering that HT is the most prevalent AID and SBI is a very popular procedure worldwide. It might be that the symptoms of possible ASIA are not assessed properly or are underestimated in women with HT leading to a late referral to a rheumatologist/immunologist.

The risk factors for ASIA development have been assessed in various studies. In 2015, four groups of patients were identified with a predisposition for ASIA: those with a prior documented immune reaction to adjuvant, those with established AID, those with a history of allergic conditions and atopic disorders, and those prone to develop autoimmunity with genetic predisposition or relevant environmental trigger (21). Among a cohort of 201 patients, in 45 women with different autoimmune rheumatic diseases (ARD) and ASIA induced by SBI, a family history of ARD was present in 42%; together with tobacco smoking, this was a significant risk factor for the development of systemic sclerosis and rheumatoid arthritis (22). The authors concluded that, in addition to SBI, the other factors should be considered for AID development, including environmental factors and genetics. We hypothesize that multiple mechanisms potentially contributed to the development of ASIA in our patient: SBI itself, previously diagnosed HT, and familial autoimmunity—the patient’s mother and grandmother were diagnosed with two different AIDs. It has been found that vitamin D deficiency is associated with the presence of autoantibodies in patients with silicone implant incompatibility syndrome and is related to the presence and/or levels of antibodies in autoimmune thyroid disease (23). Considering the role of vitamin D in the development of AID, the inadequate vitamin D levels in our patient could be considered as an additional risk factor for ASIA development.

There are very few reports of HT development after SBI. Two cases of HT after SBI were described by Vyssairta et al. (24). However, cases of HT and/or subacute thyroiditis have been described after exposure to vaccines (25–27). In a review by Bragazzi et al., 52 cases of subacute thyroiditis after exposure to different adjuvants have been collected. Among those, one case was after SBI, and one case was positive for thyroglobulin (TG) and thyroid peroxidase antibodies (28). Recently, three cases of ASIA induced by the mRNA-based SARS-CoV-2 vaccination were reported, presenting as silent thyroiditis, subacute thyroiditis, and Graves diseases (29). The authors concluded that ASIA involving thyroid disorders could be considered as adverse effects of the SARS-CoV-2 vaccination in predisposed individuals. In an update of the ASIA syndrome in 2023, the characteristics of patients with 15 different autoimmune diseases related to COVID-19 vaccines were described and summarized, among which, three cases of Grave’s disease and two cases of subacute thyroiditis were present (30). The authors concluded that these autoimmune disorders are rare, but it is very important to follow these patients for the possible development of AID in the future. Few clinical reports and animal models do not reflect the existing associations between ASIA and thyroid autoimmunity and consequently may lead to the lack of awareness about this link among physicians (31).

**Figure 3:**
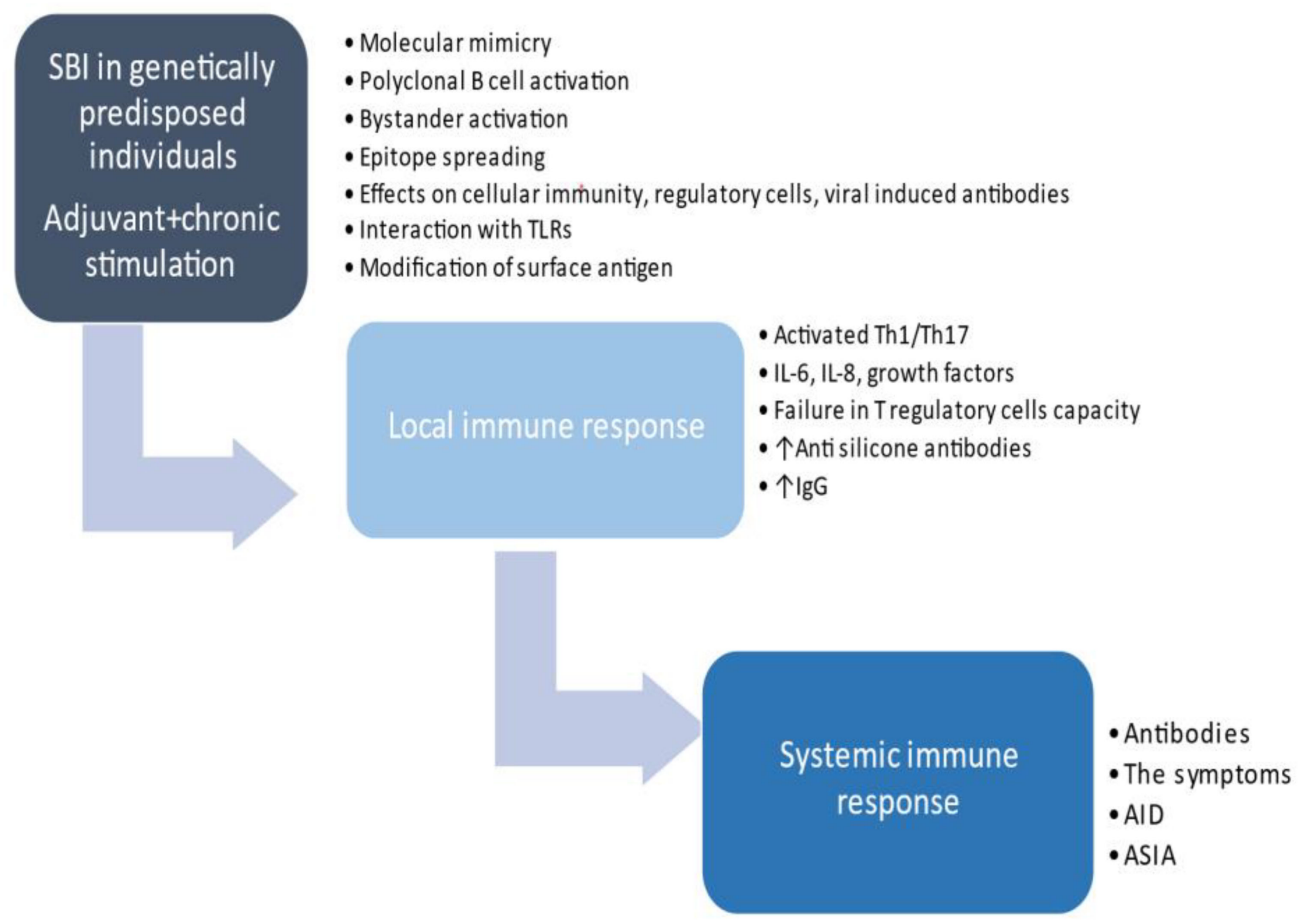
The proposed pathogenesis of autoimmune/inflammatory syndrome induced by adjuvants development in predisposed women with SBI. SBI, silicone breast implant; AID, autoimmune disease; ASIA, autoimmune/inflammatory syndrome induced by adjuvants.

Autoimmune thyroid diseases followed by SLE and rheumatoid arthritis are the most frequent diseases in familial autoimmunity (32). The existence of familial autoimmunity in patients with AID calls for awareness and a search for AID in first-degree relatives of these patients (32). Since HT is the most prevalent AID, it would be very important to identify risk factors for the development of additional AID. A large study on 495 HT patients confirmed an increased risk for additional autoimmune diseases, such as pernicious anemia, SEL, Addison’s disease, celiac disease, and vitiligo (33). It was suggested that screening for other AIDs should be done in those with autoimmune thyroid diseases who remain non-specifically unwell or develop new symptoms. Our patient with previously diagnosed HT had SBI, leading to the appearance of suggestive symptoms and diagnosis of ASIA. The patient’s symptoms have progressed over the years, and the antibodies profile changed from 2019 to 2021 (**Figure 2**). For 2 years, she was not regularly followed up and was without therapy; thus, it can be assumed that the development of new symptoms and autoantibodies probably present the natural evolution of an autoimmune process. It is postulated that, in genetically predisposed individuals, silicon may induce an adjuvant effect and chronic, persistent inflammatory stimulation and, through different and complex pathological mechanisms, may lead to a local and systemic immune response (2, 34–37). This can ultimately result in AID in some people (**Figure 3**). The presence of anti-centromere antibodies in both seroma and serum in our patient suggests that autoimmune processes are present both locally and systemically.

In addition to the growing body of research and published papers in this filed, there are no accepted or universal protocols or recommendations of pre- or post-SBI evaluation, especially in women with AID. Also, there is no consensus on whether SBI is contraindicated in women with AID. Recently, it was suggested that the patient should be evaluated for personal and familial history before SBI, and the autoantibodies should be checked in those with complaints and a positive history but not in asymptomatic patients (38). Patients with complaints and/or personal/family history of AID should undergo multidisciplinary evaluation by a plastic surgeon and a clinical consultant, and a shared decision should be made regarding SBI. The authors could not contraindicate SBI in this group of patients, but they called for attention to the development of ASIA and suggested that alternative surgical options are proposed, such as autologous tissue reconstruction and saline- or hydrocellulose-filled breast implants. Unfortunately, our patient did not receive any information regarding ASIA before SBI; thus, the lack of proper information can be viewed as a risk factor in her case. Based on the review of literature, an algorithm was made for the preoperative SBI and follow-up (39). Among other tests, preoperative determination of antithyroid antibodies was suggested as one of the markers of detection of those individuals who are prone to AID or have subclinical established AID. The authors have pointed out that the presence of antithyroid antibodies that act outside of the thyroid gland may lead to AID related to breast implants. In a recent extensive literature review by Cohen Tervaert et al., it was advised that risk factors should be assessed before SBI, especially a history of allergy, an established AID, and familial predisposition to AID (40). Plastic surgeons are advised to measure immunoglobulins preoperatively and refer patients to an internal medicine and/or rheumatologist specialist on the suspicion of abnormal clinical/and or biochemical findings.

## Conclusion

In the complex and still-not-well-understood interplay between autoimmunity and silicon in ASIA, familial autoimmunity may present the risk factor for ASIA development in women with HT. Efforts should be made to educate both patients and doctors and to raise awareness about the ASIA concept, especially in those with already present AID. Future studies are needed to establish the factors underlying the association between different AIDs in one individual and/or family members.

## Data availability statement

The original contributions presented in the study are included in the article/supplementary material. Further inquiries can be directed to the corresponding author.

## Ethics statement

Written informed consent was obtained from the individual(s) for the publication of any potentially identifiable images or data included in this article.

## Author contributions

AP, RM, MD, NK, VS, ZJ, APP, and SA analyzed and interpreted the patient data. AP and RM were major contributors in writing the manuscript. All authors contributed to the article and approved the submitted version.
